# Intracellular delivery of messenger RNA by recombinant PP7 virus-like particles carrying low molecular weight protamine

**DOI:** 10.1186/s12896-016-0274-9

**Published:** 2016-05-28

**Authors:** Yanli Sun, Yanhua Sun, Ronglan Zhao, Kunshan Gao

**Affiliations:** Institute of Nanomedicine Technology, Department of Laboratory Medicine, Institutional Key Laboratory of Clinical Laboratory Diagnostics, 12th 5-Year Project of Shandong Province, Key Discipline of Clinical Laboratory Medicine of Shandong Province, Affiliated Hospital of Weifang Medical University, Weifang Medical University, Weifang, 261053 China; Department of Hematology, Weifang People’s Hospital, Weifang, 261000 China; Department of Laboratory Medicine of Affiliated Hospital of Weifang Medical University, Weifang Medical University, Weifang, 261031 China

**Keywords:** Low molecular weight protamine, Messenger RNA, Peptide display, PP7 bacteriophage, Virus-like particle

## Abstract

**Background:**

Cell-penetrating peptides (CPPs) have been widely used as carriers to transport different molecules into living cells, whereas messenger RNAs (mRNAs) have been utilized as target molecules for the prevention and treatment of various diseases. However, the instability of CPPs and mRNAs has limited their application. Bacteriophage PP7 virus-like particles (VLPs) may protect peptides and RNAs from degradation through displaying foreign peptides on their surface and encapsidating RNA linked with the *pac site*.

**Results:**

In this study, the cDNA of the PP7 coat protein single-chain dimer carrying low molecular weight protamine (LMWP) and the cDNA of green fluorescent protein (GFP) were inserted into two multiple cloning sites of pETDuet-1, respectively. PP7 VLPs carrying the LMWP peptide and GFP mRNA were subsequently expressed in *Escherichia coli* BL21 (DE3) with high yield and thermal stability, and were easily purified. The VLPs were also non-replicative, non-infectious, and non-toxic. Moreover, they penetrated the mouse prostate cancer cells RM-1 after 24 h incubation. Last, PP7 VLPs carrying the LMWP could encapsidate the GFP mRNA, which was translated into mature protein in mammalian cells.

**Conclusions:**

Recombinant PP7 VLPs can be used simultaneously as a targeted delivery vector for both peptides and mRNA due to their abilities to package RNA and display peptides.

**Electronic supplementary material:**

The online version of this article (doi:10.1186/s12896-016-0274-9) contains supplementary material, which is available to authorized users.

## Background

Cell-penetrating peptides (CPPs) are short positively charged peptides containing less than 30 amino acid residues. They exhibit low toxicity and have the capacity to cross cellular membranes by energy-dependent and/or independent mechanisms [1, 2]. In the process, CPPs can transport different molecules such as nanoparticles [3], liposomes [4], proteins [5], peptides [5], and oligonucleotides [5–7] into living cells. Different delivery strategies such as direct conjugation, co-incubation, and non-covalent or covalent cross-linking of CPPs with target molecules [4–9] have been investigated so far.

Several factors currently limit the preclinical and clinical development of CPPs. Fist, CPPs require custom synthesis, are expensive, and can be easily degraded by intracellular or extracellular proteases [10]. Second, the crosslinking efficiency of CPPs with target molecules is low [11]. Therefore, a simple, safe, effective, stable, and cost-effective delivery vehicle for CPPs is needed to improve their stability and utilization efficiency.

Bacteriophage PP7 virus-like particles (VLPs) may be a promising tool in meeting the above requirements as a CPP delivery vehicle because PP7 VLPs can be expressed in *Escherichia coli (E. coli)* strains such as CSH41F [12]. Additionally, the PP7 coat protein has the ability to self-assemble into VLPs in the absence of viral RNA, and these VLPs in turn can serve as a vector for peptides [12, 13]. Specifically, in the viral capsid, the β-hairpin structures at the N terminus of the coat protein subunits protruding from the surface of PP7 VLPs can tolerate foreign peptides [12]. These peptides can be inserted between amino acid residues 10 and 11 of the second subunit of the coat protein single-chain dimer [12]. The insertion of DNA oligonucleotides at this site allows the production of chimeric PP7 coat proteins that have the foreign peptide placed in the central part of the hairpin. Most importantly, the displayed peptides are highly immunogenic [14]. Furthermore, the PP7 VLPs with the inserted peptides are heat-resistant up to about 70 °C and are therefore more stable than MS2 VLPs that resist heat up to 50 °C [15].

To date, the PP7 VLPs have been used as peptide vaccine carriers against the human papillomavirus [16] and malaria parasite [17]. However, whether CPPs can also be successfully displayed on the surface of PP7 VLPs and additionally retain their cell-penetrating function have not been examined yet. It is also known that PP7 VLPs encapsidate coat protein-specific messenger RNA (mRNA) [12]; however, whether they can encapsidate foreign mRNA has not been investigated either. Therefore, in this study, we have chosen the low molecular weight protamine (LMWP) (VSRRRRRRGGRRRR) peptide as the model CPP and analyzed the expression of PP7 VLPs carrying the LMWP in *E. coli* strain BL21 (DE3). We also evaluated the cell-penetrating ability of the inserted LMWP and analyzed whether PP7 VLPs could package the foreign mRNA.

## Results

### Insertion of the LMWP peptide into the PP7 coat protein dimer

**Fig. 1:**
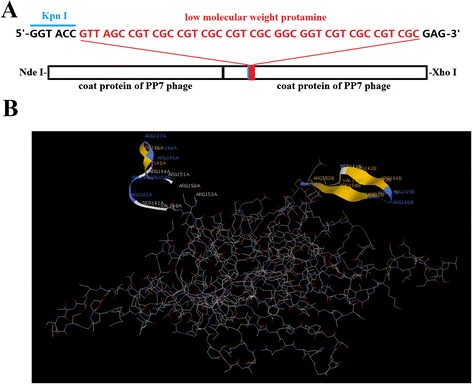
Insertion of the LMWP peptide into the PP7 coat protein dimer. The cDNA sequence of LMWP (*in red*) was inserted after the *Kpn*I site of the vector pETDuet-2PP7 (**a**). The possible structure of the PP7 coat protein dimer carrying LMWP with the AB-loops shown in ribbon was predicted using the program RasMol 2.7.1 (**b**)

The expression vector of PP7 VLPs carrying the LMWP peptide was constructed as follows: the 771-bp coding sequence of 2PP7 with one introduced restriction enzyme site *Kpn*I (Fig. 1a) was first inserted between the *Nde*I and *Xho*I restriction sites of the prokaryotic expression vector pETDuet-1. The obtained vector was named pETduet-2PP7; it encodes for a 27.944 kDa protein with 256 amino acid residues. The cDNA of LMWP was then inserted after the *Kpn*I site (Fig. 1a), which is located in the second AB-loop of the PP7 coat protein and can tolerates peptide insertions [12]. The resulting constructed vector was named pETDuet-2PP7-Protamine. All positive clones were screened by colony PCR and verified by sequencing.

To predict whether the PP7 coat protein could tolerate the insertion of LMWP peptide, the program RasMol 2.7.1 was used. The results showed the PP7 coat protein could indeed tolerate the insertion of the LMWP peptide. The secondary structure of LMWP after having been inserted in the PP7 coat protein was mainly β-sheet and β-turn (Fig. 1b).

Last, the plasmid pETDuet-2PP7-Protamine-GFP was constructed by inserting the *GFP* gene between the *Xba*I and *Bam*HI restriction sites of the plasmid pETDuet-2PP7-Protamine. The correct construct was also verified by sequencing.

### Expression of recombinant 2PP7-Protamine-GFP VLPs in *E. coli*

**Fig. 2:**
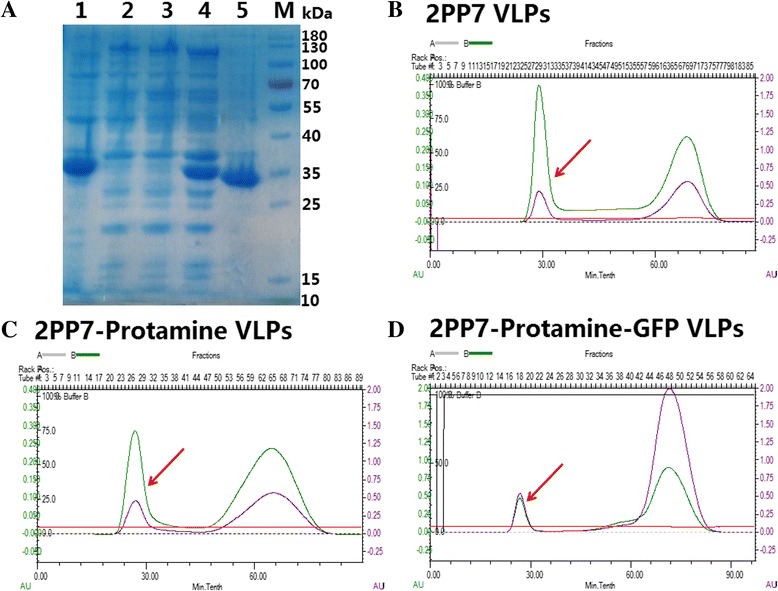
Expression of recombinant 2PP7-Protamine-GFP VLPs in *E. coli*. **a** Expression of 2PP7-Protamine and 2PP7-Protamine-GFP VLPs in the supernatant of BL21 (DE3) after sonication. Lanes of BL21 (DE3) carrying each plasmid are designated as follows: lane 1, plasmid pETDuet-2PP7-Protamine-GFP with IPTG added; lane 2, plasmid pETDuet-2PP7-Protamine without IPTG added; lane 3, pETDuet-2PP7-Protamine without IPTG added; lane 4, pETDuet-2PP7-Protamine with IPTG added; lane 5, pETDuet-2PP7 with IPTG added. **b**-**d** Purification of the VLPs. The peaks of the target proteins are marked by red arrows

To analyze the expression of PP7 VLPs carrying the peptide or not carrying the peptide, the three recombinant expression vectors above were transformed into *E. coli*. The results showed three recombinant VLPs: 2PP7 VLPs, 2PP7-Protamine VLPs, and 2PP7-Protamine-GFP, each expressed intracellularly from the plasmids pETDuet-2PP7, pETDuet-2PP7-Protamine, and pETDuet-2PP7-Protamine-GFP respectively. The above VLPs represented a dominant protein species in the supernatant of lysed bacteria after centrifugation (Fig. 2a). Furthermore, 2PP7 coat protein, 2PP7-Protamine coat protein, and 2PP7-Protamine-GFP coat protein were found in the precipitate, but whether these could fold into VLPs was not determined. SDS-PAGE analysis revealed that the molecular weight of the single-chain dimer of PP7 phage coat protein carrying the LMWP or not carrying the LMWP was approximately 33 kDa, and the latter was smaller than the former (Fig. 2a).

**Fig. 3:**
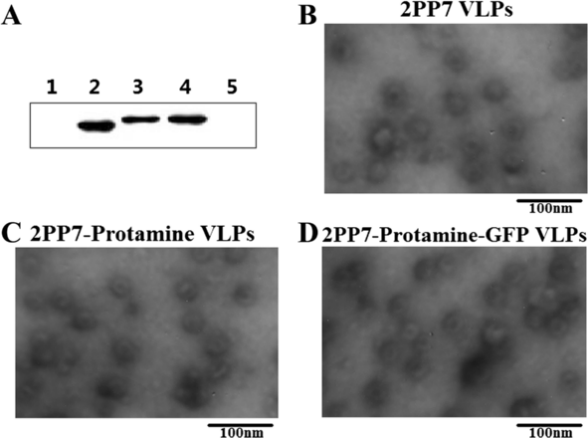
Recognition and assembly of the PP7coat protein dimer carrying LMWP into whole VLPs. **a** Western blot of VPLs: lane 1, TBS; lane 2, 2PP7 VLPs; lane 3, 2PP7-Protamine VLPs; lane 4, 2PP7-Protamine-GFP VLPs; Lane 5, BL21 (DE3) carrying pETDuet-1 induced by IPTG. **b**-**d** VLPs observed using TEM at 60 kV and 100,000× screen magnification

The VLPs were then purified by size-exclusion chromatography (Fig. 2b). The western blotting results showed the VLPs existed in the first elution peak (Figs. 2b and 3a), and the expression levels of the purified 2PP7 VLPs, 2PP7-Protamine VLPs, and 2PP7-Protamine-GFP VLPs extracted from the supernatant of 1 L of overnight culture after sonication were approximately 5.56, 2.42, and 1.45 mg/L, respectively. These results demonstrated that PP7 VLPs carrying LMWP or not carrying LMWP could be expressed in *E. coli* BL21 (DE3) with high-efficiency; however, the fusion of the peptide to PP7 coat protein and the inserted *GFP* gene in the second multiple cloning sites (MCSs) of the plasmid pETDuet-2PP7-Protamine decreased the expression of the PP7 VLPs.

### Assembly of the PP7 coat protein dimer carrying the LMWP peptide into whole VLPs

To confirm that the purified products successfully assembled into PP7 VLPs, western blotting and TEM were performed. The western blotting results showed only one specific band appearing in every lane, which demonstrated that all three recombinant VLPs could be detected by the murine antibody of the anti-coat protein of PP7 phage (Fig. 3a). In addition, the TEM results indicated that the recombinant PP7 capsids could assemble into the whole virus-like particles with a diameter of about 30 nm (Fig. 3b-d), which was similar to that of wild-type PP7 VLPs.

### Packaging and protection from degradation of the RNA linked with PP7 *pac site* by the recombinant PP7 VLPs carrying the LMWP peptide

**Fig. 4:**
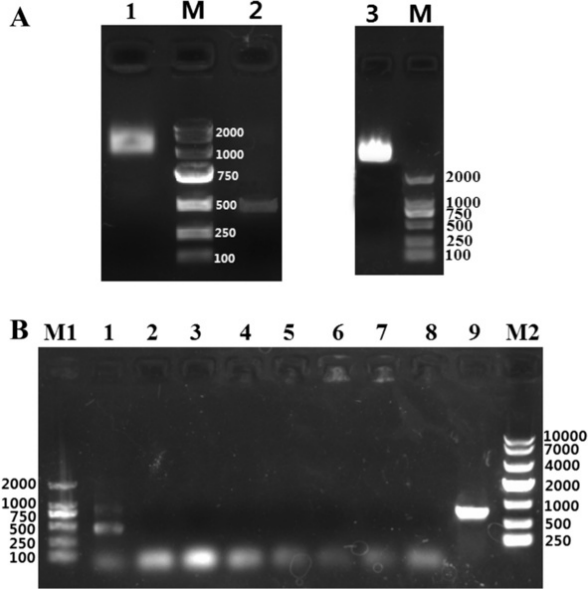
Nuclease-resistance assay of mRNA packaged by the PP7 capsid carrying LMWP. **a** The purified 2PP7 VLPs, 2PP7-Protamine VLPs and 2PP7-Protamine-GFP VLPs (800 μg/ml) were separately mixed with DNase I (20 U/mL) and RNase A (10 mg/mL), incubated at 37 °C for 12 h, and verified by 1 % agarose gel electrophoresis: lane M, DL 2000 DNA marker; lane 1, 2PP7-Protamine VLPs; lane 2, 2PP7-Protamine-GFP VLPs; lane 3, 2PP7 VLPs. **b** Verification of nucleic acids packaged by 2PP7-Protamine VLPs (lanes 1, 2, 3, 4; the PP7 coat protein dimer gene containing LMWP was amplified) and 2PP7-Protamine-GFP VLPs (lanes 6, 7, 8, 9; the GFP gene was amplified). The PCR products in different lanes were as follows: lanes 1 and 9, reverse transcription product of RNA extracted from corresponding VLPs; lanes 2 and 8, RNA extracted from corresponding VLPs; lanes 3 and 7, purified VLP sample; lanes 4 and 6, reverse transcription product of purified VLPs sample; lane 5, ddH_2_O. M1, DL 2000 DNA marker; M2, DL 10000 DNA marker

The chromatogram showed nucleic acid was present in the first peak, indicating that nucleic acids were packaged by the PP7 VLPs (Fig. 2b). To verify this result and examine the type of nucleic acid (DNA or RNA) that was packaged by the PP7 VLPs, a nuclease-resistance assay and RT-PCR were performed. Results from the nuclease-resistance assay showed that, after incubation of 2PP7 VLPs, 2PP7-Protamine VLPs, and 2PP7-Protamine-GFP VLPs with DNase I and RNase A, a band was observed in each lane but at different locations (Fig. 4a). These results showed that nucleic acids with different characteristics in terms of lengths and folding degrees were packaged by the PP7 VLPs.

Following the above assay, the DNAs or RNAs packaged by these VLPs were further verified by RT-PCR. The results showed that the RNA and not DNA was the nucleic acid packaged by these VLPs (Fig. 4b). Moreover, the RNAs most likely packaged by the 2PP7-Protamine VLPs and 2PP7-Protamine-GFP VLPs were the mRNAs of PP7 coat protein carrying LMWP and GFP (Fig. 4b). The results indicated that the PP7 VLPs carrying LMWP retained the ability to package mRNA and therefore could be used as a delivery vector of mRNA.

### Cytotoxicity and thermal stability of 2PP7-Protamine and 2PP7-Protamine-GFP VLPs

**Fig. 5:**
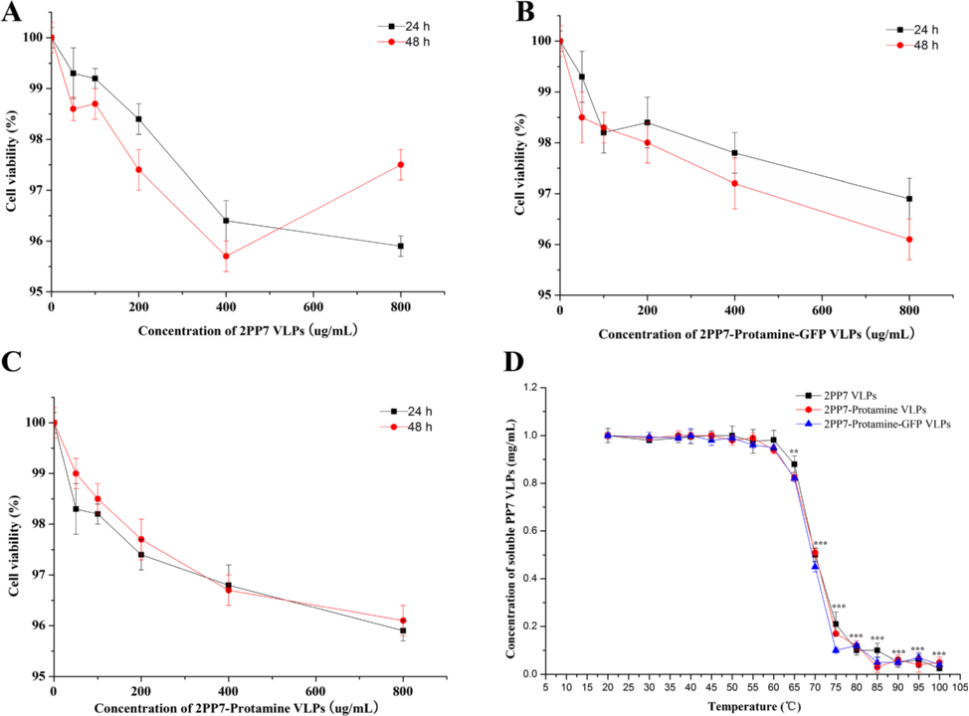
Cytotoxicity and thermal stability of 2PP7-Protamine and 2PP7-Protamine-GFP VLPs. 2PP7-Protamine and 2PP7-Protamine-GFP VLPs show low cytotoxicity in Cos-7 cells (**a**-**c**) and high thermal stability at different temperatures (**d**). The cytotoxicity has been expressed in terms of cell viability

The cytotoxicity of 2PP7, 2PP7-Protamine, and 2PP7-Protamine-GFP VLPs was examined in the African green monkey kidney fibroblast cell line Cos-7 using CCK-8 assay. The results showed no dose-dependent cytotoxicity in these three VLPs (Fig. 5a-c). Even at a high concentration of 800 μg/mL of VLPs, cell viability was higher than 95 %, demonstrating low cytotoxicity of recombinant 2PP7, 2PP7-Protamine, and 2PP7-Protamine-GFP VLPs.

At the same time, the thermal stability of these three VLPs was determined by measuring the protein concentration at different temperatures. The results showed that the concentrations of 2PP7, 2PP7-Protamine and 2PP7-Protamine-GFP VLPs decreased significantly at above 60 °C (*P* < 0.01, Fig. 5d), indicating that they resisted heating up to about 60 °C. Additionally, the stability of these three VLPs was identical at the same temperature (Fig. 5d). The results showed the high thermal stability of the PP7 VLPs carrying LMWP and of those not carrying LMWP.

### Cell-penetrating ability of PP7 VLPs carrying the LMWP peptide

**Fig. 6:**
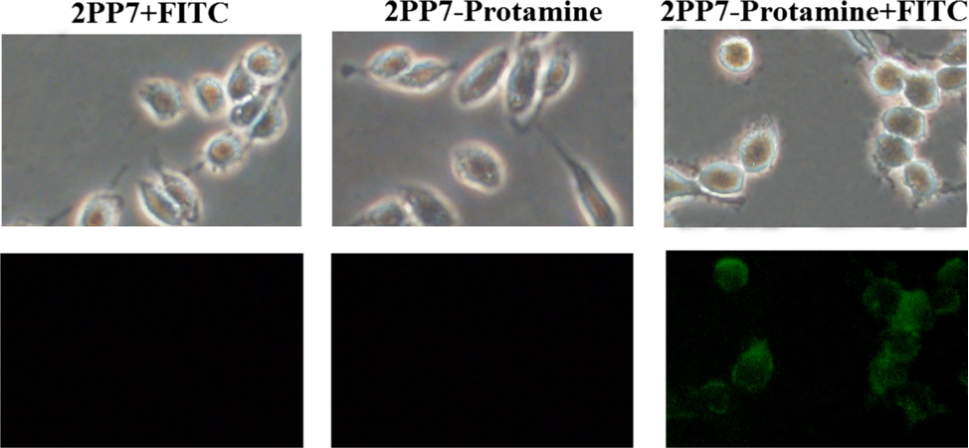
Cell-penetrating ability of PP7 VLPs carrying the LMWP peptide. Strong cell-penetrating ability was observed. Pictures were taken under white light or fluorescence (200×)

To explore whether recombinant PP7 VLPs carrying LMWP can penetrate cells, the 2PP7-Protamine VLPs were labeled with fluorescein isothiocyanate (FITC) and incubated with cells from the mouse prostate cancer cell line RM-1. After 12 h, the RM-1 cells incubated with 2PP7-Protamine VLPs exhibited a high level of fluorescence (Fig. 6). In contrast, no fluorescence could be detected in the cells incubated with unlabeled 2PP7-Protamine VLPs or FITC-labeled 2PP7 VLPs (Fig. 6). These results indicate that the LMWP was expressed on the surface of 2PP7-Protamine VLPs and that these VLPs retained strong cell-penetrating ability. Therefore, we conclude that display of other targeting cell-penetrating peptides on the surface of PP7 VLPs will be possible. The strong cell-penetrating ability of the PP7 VLPs may enhance the *in vivo* delivery efficiency of RNA and lay the foundation for targeted delivery of RNA.

### Protein translation of the mRNA packaged by PP7 VLPs carrying the LMWP peptide

**Fig. 7:**
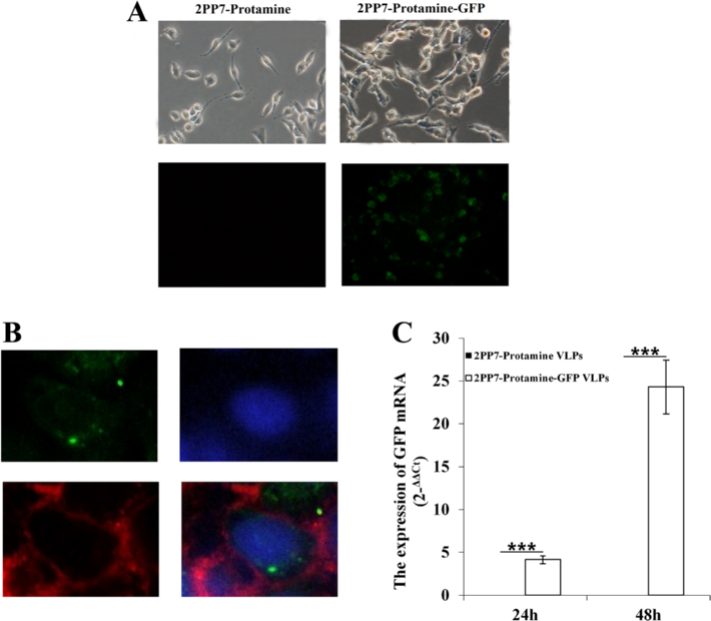
Translation of the mRNA packaged by PP7 VLPs carrying the LMWP peptide. The expression of GFP in the RM-1 cells incubated with 2PP7-Protamine VLPs or 2PP7-Protamine-GFP VLPs was observed by fluorescence microscopy (**a**) or confocal laser scanning microscopy (**b**). The levels of GFP mRNA in the cells was verified by qPCR (**c**)

We investigated whether the GFP mRNA packaged by the PP7 capsid carrying the LMWP peptide could be translated into mature protein in RM-1 cells. After 24 h of incubation with 2PP7-Protamine-GFP VLPs, most of the RM-1 cells incubated with 2PP7-Protamine-GFP VLPs exhibited green fluorescence (Fig. 7a), which was identified by the confocal laser scanning microscopy. As shown in Fig. 7b, green fluorescence was observed in the cytoplasm of almost all RM-1 cells, while red fluorescence from red fluorescent reactive dye was observed on the membrane of RM-1 cells and blue fluorescence from DAPI inside the nuclei.

Additionally, the levels of GFP mRNA level in the RM-1 cells were verified by quantitative real-time PCR (qPCR) at 24 h and 48 h (Fig. 7c). As shown in Fig. 7c, whether at 24 h or at 48 h, the GFP mRNA could not be detected in the cells incubated with 2PP7-Protamine VLPs. Instead, the higher level of GFP mRNA was observed in thecells incubated with 2PP7-Protamine-GFP VLPs (*P* < 0.001). Moreover, in this group, the level of GFP mRNA at 48 h was significantly higher than that at 24 h. All these results showed that PP7 VLPs carrying the LMWP peptide could penetrate cell membrane and that the mRNA packaged by them was translated into mature protein in mammalian cells.

## Discussion

Molecules such as mRNAs have been used for the prevention and treatment of various diseases [18–21], including cancer [18–20]. However, there are several obstacles in the *in vivo* application of mRNAs, including the low *in vivo* delivery efficiency of mRNA, their lack of cell-type specificity, and instability. In this study, we introduced a delivery system based on PP7 VLPs and a cell-penetrating peptide as a solution to the above limitations of mRNA-targeted delivery.

In order to improve the delivery efficiency of mRNA, the PP7 VLPs were decorated with a non-toxic cell-penetrating peptide, LMWP. This peptide, similar to the peptide TAT, enhanced the intracellular delivery of the linked particles without any dependence on receptors, temperature conditions, or energy use and without causing any alterations to the cell membrane [22–24]. The cell-penetrating assay showed that the LMWP peptide was displayed on the surface of PP7 VLPs.

Previous studies have shown that MS2 capsids specifically encapsidate specific mRNAs that are linked to the MS2 *pac site* [25–27]*,* and our results showed PP7 capsids could also do this. As was expected, we found that PP7 VLPs carrying the cell-penetrating peptide LMWP packaged a specific mRNA linked with the *pac site* and protected the RNA from rapid extracellular degradation by RNase. Furthermore, the PP7 VLPs carrying the cell-penetrating peptide LMWP and GFP mRNA efficiently penetrated the cell membrane. To date, the greatest length of RNA packaged using one wild-type *pac site* in MS2 VLPs fused with a peptide was 970 bases [28]; the length of the RNA using the PP7 VLPs was 720 bases. However, it has not been determined whether more than one kind of RNA was packaged by the PP7 VLPs during their expression in *E. coli*. The size and characteristics of the foreign RNA that were packaged by the peptide fused to the PP7 coat protein will therefore need to be studied further.

In addition, the mRNA packaged by the PP7 VLPs showed a high level of protein expression in mammalian cells within 24 h. This result was not in accordance with that observed for the naked mRNA vaccine, which showed maximum expression within 12–18 h [29, 30]. This finding underscores the high stability of PP7 VLPs carrying the LMWP peptide.

In 2011, Caldeira and Peabody proved that PP7 VLPs with a peptide insertion were generally stable up to about 70 °C [15]. In this study, the PP7 VLPs carrying the cell-penetrating peptide LMWP and the GFP mRNA showed similar thermal stability up to about 60 °C as those carrying other peptides and, in addition, were more stable than the MS2 VLPs with peptide insertion.

In summary, our current study demonstrates that PP7 VLPs carrying a cell-penetrating peptide and mRNA can be expressed in *E. coli* with high efficiency, are easily purified, and are nuclease-resistant, non-replicative, nontoxic, and noninfectious.

## Conclusions

The current study provides a stable and efficient mRNA delivery system that is based on the inherent abilities of PP7 VLPs to encapsidate mRNA and tolerate foreign peptides. Moreover, the recombinant PP7 VLPs carrying the CPP and mRNA can be easily expressed in a prokaryotic expression system at high yield, are nuclease-resistant, non-replicative, nontoxic, and non-infectious. In addition, the mRNA packaged by these VLPs showed a high level of protein expression in mammalian cells. In conclusion, this system holds great potential in the development of mRNA and peptide-based targeted therapeutics to be used for the prevention and treatment of cancer and other diseases.

## Methods

### Plasmid construction

The single-chain dimer gene of PP7 phage coat protein (accession number: NC_001628.1) with one *Kpn*I site was amplified by the overlap extension PCR, and inserted into restriction enzyme sites *Nde*I and *Xho*I of the vector pETDuet-1 (Novagen, Nottingham, UK). This constructed plasmid was named as pETDuet-2PP7, which was finally verified by sequencing analysis (Genscript, Nanjing, China).

The plasmid pETDuet-2PP7-Protamine was constructed based on the plasmid pETDuet-2PP7. Briefly, the gene containing LMWP was amplified by PCR using primers 2PP7-Protamine-for and PP7-r (Table 1), digested by two restriction enzymes *Kpn*I and *Xho*I (Takara, Dalian, China), and ligated with the plasmid pETDuet-2PP7. After that, the plasmid pETDuet-2PP7-Protamine-GFP was constructed with GFP DNA (accession number: NC_025025.1) inserted between *Xba*I and *Bam*HI of the plasmid pETDuet-2PP7-Protamine. The correct construct was verified by sequencing analysis. The sequence of all primers are listed in Table 1.

**Table 1:** Primers and synthesized oligonucleotides

Primer	Sequence(5’-3’)
PP7-for	5’-GGAATTCCATATGTCCAAAACCATCGTTC-3’
GFP-for	5’-GCTCTAGAGCCGCCATGGTGAGCAAGGGCGAGGA-3’
GFP-r	5’-CGGGATCC *TAAGGGTTTCCATATAAACTCCTTA*TCATTACTTGTACAGCTCGTCCA-3’
PP7-r	5’-CCGCTCGAGTTAACGGCCCAGCGGCAC-3’
2PP7-Protamine-for	5’-GGGGTACC **GTTAGCCGTCGCCGTCGCCGTCGCGGCGGTCGTCGCCGTCGC**GAGGCAACCCGCACTCTG-3’
GFP-qRT-for	5’-GCAGAAGAACGGCATCAA-3’
GFP-qRT-r	5’-GGTGCTCAGGTAGTGGTTGT-3’
GAPDH-for	5’-AAATGGTGAAGGTCGGTG-3’
GAPDH-r	5’-GTGAGTGGAGTCATACTGGAAC-3’

Restriction enzyme sites Nde I, Kpn I and Xho I are underlined; the cDNA sequence of LMWP is in boldface; *pac site* is in italic type

### Protein expression and purification

The plasmids mentioned above were isolated from 2 mL overnight cultures of single colonies using a QIAprep Spin Plasmid Kit (Qiagen) and separately transformed into the competent BL21 (DE3) cells. The transformed cells were cultured in 5 ml of LB medium supplemented with ampicillin (50 mg/L). The precultures were used to inoculate 500 mL of LB medium supplemented with ampicillin. When these cultures were grown to an OD_600_ of approximately 0.6 at 37 °C, isopropyl thio-β-D-galactoside (IPTG) was added with a final concentration of 1 mM and these cultures were incubated overnight at 37 °C. The cells were harvested using centrifugation at 12,000 rpm for 1 min at 4 °C, resuspended in a final volume of 30 mL TBS (10 mM Tris–HCl, 100 mM NaCl, 1 mM MgCl_2_, 0.1 mM EDTA, pH 7. 4), and disrupted by sonication (JP96-II, Shanghai, China) on ice 200 times (*on* for 3 s, *off* for 6 s). The homogenate was centrifuged at 12,000 rpm, 4 °C for 30 min. The supernatant was collected, and 30 mL of chloroform was added and mixed with the solution. The mixture was centrifuged at 12,000 rpm, 4 °C for 15 min, and the supernatant was collected. The nanoparticles in the supernatant were analyzed by sodium dodecyl sulfate-polyacrylamide gel electrophoresis (SDS–PAGE) on 12 % gels. Then the supernatant was incubated with DNase I (20 U/mL) and RNase A (10 mg/mL) (Sigma, St Louis, MO) at 37 °C for 3 h, and purified by Bio-Gel A-1.5 m gel (Bio-Rad, USA) filtration chromatography (BioLogic DuoFlow QuadTec system, Bio-Rad, USA).

### Western blotting

The purified products were verified by western blotting. The purified VLPs was transferred from 12 % SDS-PAGE gels onto a 0.45 μm polyvinylidene fluoride (PVDF) membrane in the buffer containing 25 mM Tris-HCl (pH 8.3), 192 mM glycine, 20 % methanol, and blocked with 5 % fat-free dry milk in phosphate-buffered saline (PBS) for 1 h. These membranes were incubated with anti-PP7 coat protein (prepared using wild-type PP7 phage coat protein as the immunogen by our lab) overnight at 4 °C and with HRP-labeled secondary antibodies (Univ-bio, China) at 37 °C for 1 h in order. The signal was detected using the Image Lab software (Bio-Rad, USA).

### Transmission electron microscopy (TEM)

The recombinant PP7 VLPs were adsorbed on carbon-coated glow-discharged copper grids for 2 min, and were negatively stained with 2 % uranyl acetate for 2 min. The VLPs were visualized using a Hitachi H7500 (Japan) transmission electron microscope at a magnification of 100,000 × .

### Nuclease resistant assay

The purified 2PP7 VLPs, 2PP7-Protamine VLPs and 2PP7-Protamine-GFP VLPs (800 μg/ml) were separately mixed with DNase I (20 U/mL) and RNase A (10 mg/mL), incubated at 37 °C for 12 h, and verified by 1 % agarose gel electrophoresis.

### Identification of nucleic acid packaged by PP7 capsids using PCR

RNA isolation was performed using TRIzol (Thermo Fisher Scientific, MA, USA) according to the manufacturer’s instructions. To identify of the nucleic acid packaged by 2PP7-Protamine VLPs and 2PP7-Protamine-GFP VLPs, respectively, ordinary PCR or reverse transcription-polymerase chain reaction (RT-PCR) was performed with the appropriate primers and templates at 94 °C for 5 min, followed by 30 cycles of 10 s at 98 °C, 30 s at 60 °C, and 60 s at 72 °C, and followed by 7 min at 72 °C. 2PP7 gene and GFP gene were amplified using primers PP7-for paired with PP7-r and GFP-for paired with GFP-r, respectively. The PCR products were verified by 1 % agarose gel electrophoresis with ethidium bromide staining, then purified and ligated into the pMD18-T plasmid (Takara, Dalian, China) for further verification by sequencing.

### Cytotoxicity assay

Cos-7 cells (1 × 10^4^/well) were incubated with various concentrations of PP7 VLPs (0, 50, 100, 200, 400, 800 μg/mL) for 24 h or 48 h in triplicate. 10 μL CCK (cell counting kit)-8 solution (Dojindo, Mashikimachi, Kumamoto, Japan) was carefully added to each well 2 h before detection. The absorbance of each well was measured at 450 nm with an ELISA plate reader (Multiskan Go, Thermo, USA).

### Cell penetrating assay

The 2PP7 VLPs and 2PP7-Protamine VLPs were fluorescently labeled with fluorescein isothiocyanate (FITC, Sigma, St Louis, MO, USA), according to the manufacturer’s instructions. Briefly, 50 μL of 1 mg/mL FITC was mixed with 950 μL of 2 mg/mL 2PP7 or 2PP7-Protamine VLPs in a dialysis bag (MW: 3500, Union Carbide, USA). The mixture was dialyzed in 0.1 M carbonate-bicarbonate buffer (pH 9.0) at 4 °C in darkness for 12 h. After that, the mixture was dialyzed in 2 L of PBS at 4 °C for 12 h. The FITC-labeled 2PP7-Protamine VLPs were purified using Sephacryl S-200 gel exclusion chromatography. Following that, the purified VLPs were added to 1 × 10^6^ RM-1 cells per well with a final concentration of 10 μg/mL. Unlabeled 2PP7-Protamine VLPs and labeled 2PP7 VLPs were used as negative controls. After 12 h of incubation, the cells were washed three times with PBS to remove the VLPs-containing medium. Intracellular distribution of the VLPs was monitored under 200× magnification using a fluorescence microscope with an excitation filter BG12 and an absorption filter OG4.

### Thermal stability assay

The thermal stability of VLPs was determined by measuring the fraction of proteins that remained soluble after 2 min at a given temperature [15]. 25 μL of VLP solutions at a concentration of 1 mg/mL in TBS were added to preheated tubes at 20, 30, 37, 40, 45, 50, 55, 60, 65, 70, 75, 80, 85, 90, 95, 100 °C. After 2 min, the tubes were transferred into ice, where they remained for 8 min until they were subjected to centrifugation at 18,000 g, 4 °C for 5 min in microcentrifuge tubes. The supernatant fraction was then transferred into fresh tubes. Using bicinchoninic acid assay, the amount of proteins in the soluble fractions was determined [31]. The values shown in Fig. 5d are the averages of three independent measurements.

### Expression of packaged exogenous messenger RNA (mRNA) in mammalian cell line

The 2PP7-Protamine-GFP VLPs (100 nM/mL) were added to 3 × 10^4^ RM-1 cells in 24-well plates in triplicate. After 24 h, the expression of GFP protein was monitored firstly by a fluorescence microscope. Then the cells were stained with red fluorescent reactive dye (L23102, Life Invitrogen, USA) and DAPI (Beyotime, China) in order before detection. The expression of GFP protein was further monitored by confocal laser scanning microscopy (LSM510 laser scanning microscope, Carl Zeiss, Germany).

### Quantitative real-time PCR (qPCR)

In a six-well plate, 1 × 10^5^ cells/well was cultured. The 2PP7-TAT-23b or 2PP7-TAT-con VLPs (100nM) were then added to each well and incubated with the cells for 24 h or 48 h at 37 °C. Total RNA was extracted from the harvested cells with TRIzol. For GFP mRNA detection, the primers were designed on the base of these sequences, as previously described (Table 1). The expression levels of GFP mRNA were quantified by real-time PCR with the PrimeScript RT reagent Kit and SYBR Premix Ex Taq™ II Kit (Takara, Dalian, China). Briefly, approximately 50 ng of mRNA from each sample was reverse-transcribed to cDNA with the GFP-qRT-r primer and the GAPDH-r primer. Real-time PCR was then performed on a StepOnePlus^TM^ Real-Time PCR System. All reactions were run in duplicate. In this study, GAPDH mRNA was chosen as the internal control of GFP mRNA. The relative expression levels of microRNAs were calculated with the 2^-⊿⊿Ct^ method [32], and the differences in GFP mRNA concentrations between the treated and control groups were expressed as fold changes.

### Statistical analysis

Statistical analyses were performed using the GraphPad Prism Software (version 5.01). Data were considered statistically significant when *P* < 0.05.

## Additional file

Additional file 1: Figure S1. 2PP7-Protamine-GFP VLPs package the mRNAs of GFP or PP7 coat protein carrying LMWP. The templates of PCR in different lanes were as follows: lanes 1 and 6, purified VLPs sample; lanes 2 and 5, RNA extracted from related VLPs; Lanes 3 and 4, reverse transcription product of RNA extracted from related VLPs. M, DL 2000 DNA marker. **Figure S2.** The distribution of 2PP7-Protamine-GFP VLPs in the RM-1 cells. The black arrow indicates the location of 2PP7-Protamine-GFP VLPs. **Figure S3.** The sketch of structure of 2PP7-Protamine-GFP VLPs. (DOC 632 kb)

## Abbreviations

CCK: cell counting kit
CPP: cell-penetrating peptide
*E. coli*: *Escherichia coli*
FITC: fluorescein isothiocyanate
IPTG: isopropyl thio-β-D-galactoside
LMWP: low molecular weight protamine
mRNA: messenger RNA
PBS: phosphate-buffered saline
PVDF: polyvinylidene fluoride
RT-PCR: reverse transcription-polymerase chain reaction
SDS-PAGE: sodium dodecyl sulfate-polyacrylamide gel electrophoresis
TEM: transmission electron microscopy
VLP: virus-like particle
