# Supplementary material for: Intracellular delivery of messenger RNA by recombinant PP7 virus-like particles carrying low molecular weight protamine
Source: BMC Biotechnol. 2016 May 28;16:46. doi: 10.1186/s12896-016-0274-9 (PMC4884372; doi:10.1186/s12896-016-0274-9)
Supplement: Additional file 1: Figure S1. — 2PP7-Protamine-GFP VLPs package the mRNAs of GFP or PP7 coat protein carrying LMWP. The templates of PCR in different lanes were as follows: lanes 1 and 6, purified VLPs sample; lanes 2 and 5, RNA extracted from related VLPs; Lanes 3 and 4, reverse transcription product of RNA extracted from related VLPs. M, DL 2000 DNA marker. Figure S2. The distribution of 2PP7-Protamine-GFP VLPs in the RM-1 cells. The black arrow indicates the location of 2PP7-Protamine-GFP VLPs. Figure S3. The sketch of structure of 2PP7-Protamine-GFP VLPs. (DOC 632 kb) [file 12896_2016_274_MOESM1_ESM.doc]

**Additional file**

**Intracellular delivery of messenger RNA by recombinant PP7 virus-like particles carrying low molecular weight protamine**

Yanli Sun ^1^, Yanhua Sun^2^*, Ronglan Zhao^1^, Kunshan Gao^3^

^1^Institute of Nanomedicine Technology, Department of Laboratory Medicine, Institutional Key Laboratory of Clinical Laboratory Diagnostics, 12^th^ 5-Year Project of Shandong Province, Key Discipline of Clinical Laboratory Medicine of Shandong Province, Affiliated Hospital of Weifang Medical University, Weifang Medical University, Weifang 261053, China

^2^Department of Hematology, Weifang People’s Hospital, Weifang 261000, China

^3^Department of Laboratory Medicine of Affiliated Hospital of Weifang Medical University, Weifang Medical University, Weifang 261031, China


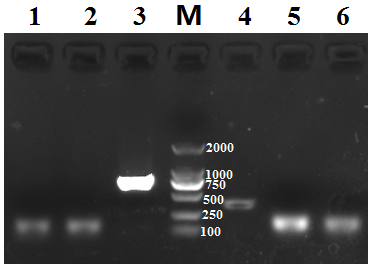


**Figure S1** 2PP7-Protamine-GFP VLPs package the mRNAs of GFP or PP7 coat protein carrying LMWP. The templates of PCR in different lanes were as follows: lanes 1 and 6, purified VLPs sample; lanes 2 and 5, RNA extracted from related VLPs; Lanes 3 and 4, reverse transcription product of RNA extracted from related VLPs. M, DL 2000 DNA marker

**
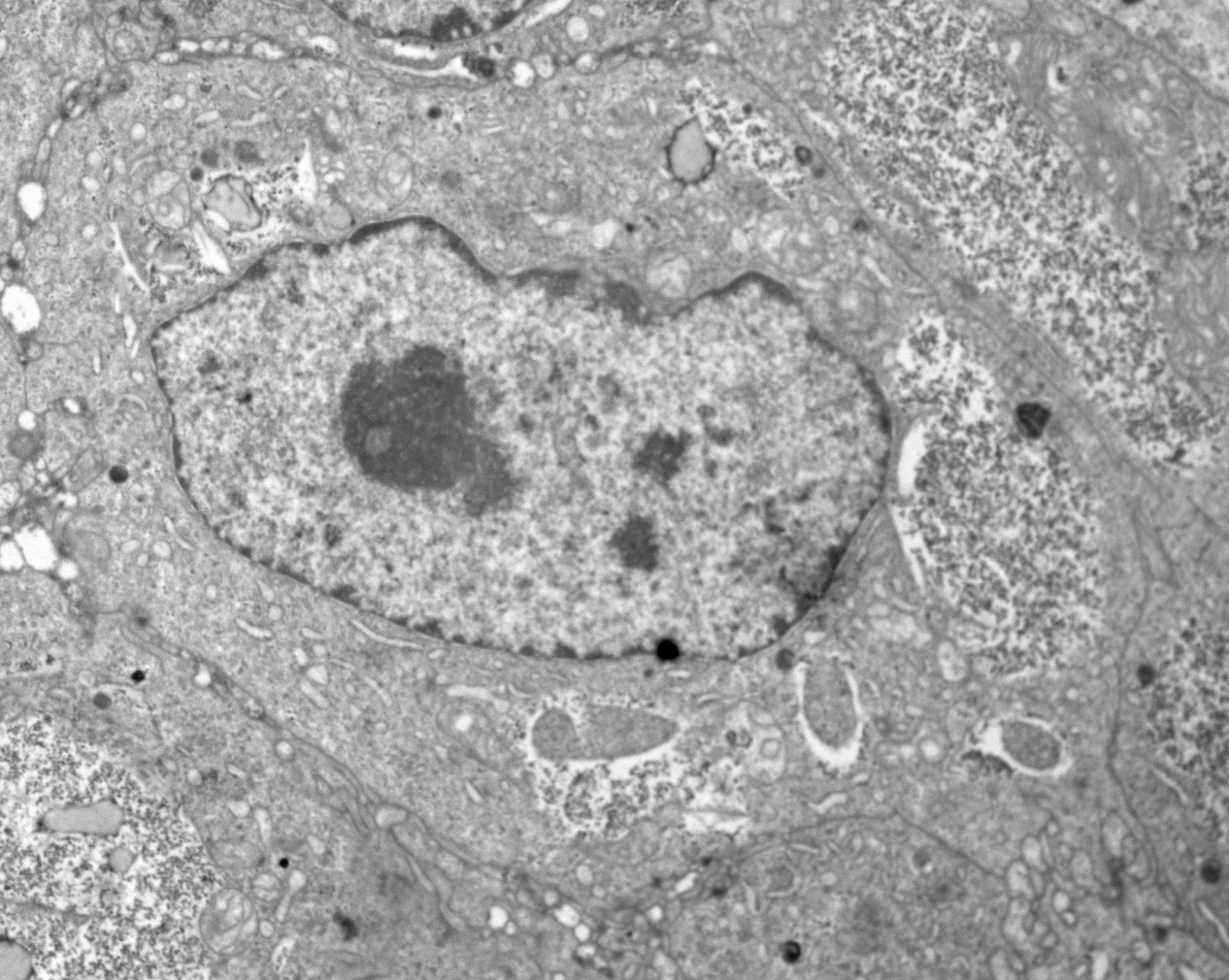
**

**Figure S2** The distribution of 2PP7-Protamine-GFP VLPs in the RM-1 cells.

The black arrow indicates the location of 2PP7-Protamine-GFP VLPs.


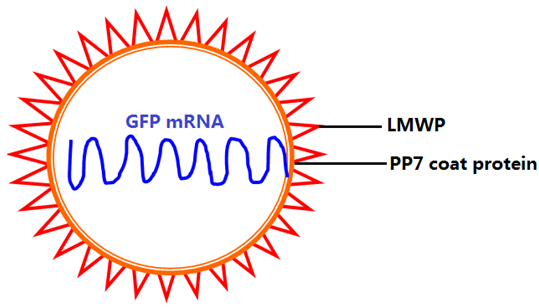


**Figure S3** The sketch of structure of 2PP7-Protamine-GFP VLPs
